# Influence of natural and non-natural diets on the fitness and rearing of *Pectinophora gossypiella* Saunders

**DOI:** 10.1038/s41598-023-40712-6

**Published:** 2023-08-22

**Authors:** Rabia Saeed, Muhammad Waqar Ul Hassan, Waqar Jaleel, Muhammad Ikhlaq, Syed Ishfaq Ali Shah, Safia Niaz, Rashid Azad, Rasheed Akbar, Zahid Mahmood, Adeel Mukhtar, Syed Muhammad Zaka, Khawaja G. Rasool, Mureed Husain, Montaser M. Hassan, Abdulrahman S. Aldawood, Muhammad Shakeel

**Affiliations:** 1 Entomology Section, Central Cotton Research Institute, Multan, Punjab 60000 Pakistan; 2 Department of Entomology, Faculty of Agricultural Sciences and Technology, Bahauddin Zakariya University, Multan, 60800 Pakistan; 3 Horticultural Research Station Bahawalpur, Multan, Punjab Pakistan; 4 Department of Agricultural Engineering, Fareed Biodiversity and Conservation Centre, Khawaja Fareed University of Engineering and Information Technology, Rahim Yar Khan, Punjab Pakistan; 5 Department of Entomology, Faculty of Basic and Applied Sciences, The University of Haripur, Haripur, Khyber Pakhtunkhwa 22062 Pakistan; 6 Institute of Environment and Ecology, School of the Environment and Safety Engineering, Jiangsu University, Zhenjiang, China; 7 Plant Protection Department, College of Food and Agriculture Sciences, King Saud University, P.O. Box 2460, 11451 Riyadh, Saudi Arabia; 8 Department of Biology, College of Science, Taif University, P.O. Box 11099, 21944 Taif, Saudi Arabia; 9 Key Laboratory of Bio-Pesticide Innovation and Application of Guangdong Province, College of Agriculture, South China Agricultural University, Guangzhou, China

**Keywords:** Ecology, Behavioural ecology, Entomology

## Abstract

In order to develop integrated management approaches for *Pectinophora gossypiella*, basic studies are crucial. The two-sex life table is the most important tool for describing the fitness and population parameters of both sexes (male and female) of an insect, while the traditional life table only explains the female sex of an insect. However, no study has reported on the biology of *P. gossypiella* using two-sex life table tools. Therefore, this study explains the rearing dynamics of *P. gossypiella* on a cotton seed-based artificial diet and a natural diet (mature cotton bolls). According to the results, the oviposition period of *P. gossypiella* was recorded to be longer on the artificial diet (9.07 ± 0.24) compared to the natural diet (7.40 ± 0.11). The total fecundity of *P. gossypiella* was greater on the artificial diet (125.94 ± 3.06) in comparison to the natural diet (60.37 ± 1.10). The population parameters, including intrinsic rate of increase, finite rate of increase, gross reproductive rate, and net reproductive rate of *P. gossypiella* were highest on the artificial diet in comparison to the natural diet. This study concluded that the cotton seed-based artificial diet was most suitable for the rearing of *P. gossypiella*. In the future, *P. gossypiella* may be studied in depth in light of the findings in this study.

## Introduction

Cotton crops are called “white gold” all over the world, and have been cultivated in more than 33 million hectares worldwide^1^. Cotton is the second most important economic crop in Pakistan and makes up 0.80% of Pakistan's GDP. Pakistan is the world’s third largest consumer of cotton, the fourth largest cotton grower, and the seventh largest apparel producer^2^. Over the last two decades, Bt crops have covered 185.1 million hectares of land^3^. The most popular use of protein engineering technology is transgenic agriculture, including Bt cotton, because it efficiently controls lepidopterous insects^4^. However, cotton yield is affected by various density-dependent and independent factors^5,6^. Insect pests, particularly *Pectinophora gossypiella* (Saunders), are the most notorious pest in cotton crop, causing 20–30% yield reduction^7^. The Bt cotton types (FH-142, MNH-886, IR-3701, FH-Lalazar) have shown resistance against pink bollworm^8^. In contrast, non-Bt refuge has caused resistance in bollworms against Bt cotton, e.g., *P. gossypiella*, Monophagous caterpillars such as *P. gossypiella*^9^ can become resistant to Bt toxins when they eat Bt cotton in their early stages (early life stages)^10,11^. The continued planting of Bt cotton creates a selection pressure for resistant caterpillars. Increasing the number of Bt cotton acres increases its ability to resist the toxin, resulting in a population that is more resistant than before. Because larvae of *P. gossypiella* are oligophagous or mostly monophagous^9^, its initial stages are more vulnerable to any detrimental effect. e.g., second instar of bollworm for insecticide bioassay. first instar cannot be used due to small size and susceptibility^10,11^. While in case of *P. gossypiella*, the 1st instar enters inside the cotton bolls from tip within 30–45 min just after hatching^9^.

The *P. gossypiella* is the most economically harmful pest and feeds on reproductive parts of cotton crops in an inconspicuous manner^12–14^. Nearly 4.5 million bales of cotton yield were lost in Pakistan due to their attacks during 2015 and 2016^15^. Furthermore, because of the oligophagous or mostly monophagous nature of feeding on cotton fruiting parts*,* the *P. gossypiella* has developed resistance against Bt-toxin^16–18^. Practice of growing non Bt cotton as refugia is not prevailing in Pakistan^19,20^. This is the main reason for resistance development in monophagous or oligophagous insects so it can develop resistance easily because genes cannot be diluted with susceptible population compared to those feed on alternative host^14,21^.

The study of basic information of insect pests is necessary for their integrated pest management (IPM)^22,23^. However, the biology and rearing of *P. gossypiella* have been reported on cotton bolls^24^, okra^25^, artificial diets including cotton seed, chickpea flours^25,26^, Bt and non-Bt cotton bolls^27^, and wheat germ^28^ via traditional life table tools. To study the basic information on artificial diet is more convenient. Meanwhile, it is challenging to employ a natural diet, e.g., cotton bolls, throughout the year in laboratory rearing of *P. gossypiella*. And artificial diet must be have sufficient quality and quantity of protein and nutrients that are essentials for the fitness and reproduction of insects^29^, in other words artificial diet must be alternate to natural host that are easily available to insect in fields. Two-sex life traits are crucial when aiming at gaining a better understanding of a pest, e.g., when the population of pest or predator is vulnerable and resistant^30–32^.

Therefore, this study explains the fitness parameters using two-sex life table tools. Due to COVID-19 issues, ingredients of the artificial diet for *P. gossypiella* have become expensive to import or purchase from developed countries, e.g., the USA and China. In our study, the cotton seed-based artificial diet was prepared for the rearing of *P. gossypiella* in the laboratory. The ingredients of this diet (Table 1) are easily available and cheaper in local markets in Pakistan. Additionally, this study would be more beneficial for laboratory and field research as well as understanding how to utilize pesticides and biological control agents when the population is at its lowest, most vulnerable, and least resistant. The biology and fitness of *P. gossypiella* on a cotton seed-based artificial diet compared with a natural diet (cotton bolls) by using two-sex life table tools are, however, lacking.

**Table 1:** Ingredients used for artificial diet of* P. gossypiella.*

Sr. no	Name of diet	Quantity of ingredients
A	Fraction A	
1	Cotton seed flour	50 g
2	Chickpea flour	100 g
3	Sucrose	15 g
4	Distilled water	200 mL
B	Fraction B	
1	Agar–agar	10 g
2	Distilled water	200 mL
C	Fraction C	
1	Dried yeast powder	8.0 g
2	Ascrobic acid	1.2 g
3	Methyl 4-Hydroxy benzoate	1 g
4	Multivitamin	4.0 mL
5	Streptomycin sulphate	0.2 g
6	Casein	10 g
7	Cystiene	0.1 g
8	Wesson salt	1 g
9	Oil	2 mL
10	Sorbic acid	1 g

## Results

### Biological parameters

There was a significant difference in egg developmental time or incubation period (time between laying and hatching) of *P. gossypiella*; it was shorter on the artificial diet (2.64 ± 0.09 d) compared to on the natural diet (4.35 ± 0.09 d), as shown in Table 2. However, there was no major difference in the first, second, third, and fourth instars’ duration on the artificial and the natural diet. Pupal duration of *P. gossypiella* was significantly (*p* < 0.0001) longer on the artificial diet (9.25 ± 0.12 d) than on the natural diet (6.91 ± 0.13 d). Female adult duration was longer than male adult duration on both the artificial and the natural diet. Furthermore, male adult longevity was greater on the artificial diet (15.83 ± 0.4 d) than on the natural diet (14.95 ± 0.43 d). Similarly, female adult lifespan was significantly higher on the artificial diet (26.58 ± 0.44 d) compared to the natural diet (20.07 ± 0.28 d). There was a visible difference in the oviposition period of *P. gossypiella*; it was longer on the artificial diet (9.07 ± 0.24 d) than on the natural diet (7.40 ± 0.11 d), as shown in Table 2. There was a significant difference in the fecundity of *P. gossypiella*: 125.94 ± 3.06 eggs/female when fed on the artificial diet and fewer eggs on the natural diet (60.37 ± 1.10 eggs/female), as clearly shown in Table 2.

**Table 2:** Stimulus of artificial and natural diets on the biological parameters of *P. gossypiella.*

Traits	Artificial diet	Natural diet	F	*t*	df
Egg (d)	2.64 ± 0.09 b (n:100)	**4.35 ± 0.09 a (n:97)**	0.97	− 13.82	195
1st instar (d)	2.69 ± 0.06 a (n:97)	2.69 ± 0.05 a (n:97)	0.17	0.00	192
2nd instar (d)	3.73 ± 0.06 a (n:93)	3.78 ± 0.06 a (n:94)	0.03	− 0.53	185
3rd instar (d)	5.66 ± 0.08 a (n:89)	5.74 ± 0.09 a (n:88)	0.25	− 0.64	175
4th instar (d)	6.28 ± 0.06 a (n:89)	6.26 ± 0.06 a (n:85)	0.49	0.25	172
Pupae (d)	**9.25 ± 0.12 a (n:85)**	6.91 ± 0.13 b (n:85)	0.06	13.43	168
Male longevity (d)	**15.83 ± 0.40 a (n:42)**	14.95 ± 0.43 b (n:40)	0.15	1.49	80
Female longevity (d)	**26.58 ± 0.44 a (n:43)**	20.07 ± 0.28 b (n:45)	5.78	12.53	86
TPOP (d)	34.74 ± 0.37 a (n:43)	34.80 ± 0.42 a (n:45)	0.10	− 0.10	86
APOP (d)	4.16 ± 0.11 a (n:43)	**4.36 ± 0.14 a (n:45)**	3.35	− 1.08	86
Oviposition (d)	**9.07 ± 0.24 a (n:43)**	7.40 ± 0.11 b (n:45)	13.04	6.43	86
Fecundity/female	**125.94 ± 3.06 a (n:43)**	60.37 ± 1.10 b (n: 45)	13.04	6.43	86

d: days**, n:** no. of individuals**,** APOP: adult pre-oviposition period of a female adult, TPOP: total pre-oviposition period of a female counted from emergence. The standard errors of the mean values of each biological parameter were estimated using two-sex life table software. The independent *t*-test was used to estimate the difference between the two treatments (artificial and natural diets) showed by different lower-case letters. And significantly higher mean values are represented in bold.

### Population Parameters

There was a small difference in the intrinsic rate of increase (r) and the finite rate of increase (*ʎ*) between the two diets. However, values of *r* and *λ* were a bit higher on the artificial diet (*r* = 0.10, *λ* = 1.11) in comparison to the natural diet (*r* = 0.09, *λ* = 1.09) (Table 3). There was a remarkable difference in gross reproductive rate (*GRR*) of *P. gossypiella* reared on the two different diets; the *GRR* was significantly greater on the artificial diet (67.54 offspring) than on the natural diet (33.49 offspring). There was also a significant difference in the net reproductive rate *R*_*0*_ (offspring/individual) of *P. gossypiella* between the artificial and natural diets; *R*_0_ was noticeably higher on the artificial diet (54.16 offspring/individual) compared to the natural diet (27.17 offspring/individual) (Table 3). There was no significant difference in the mean generation time between the artificial and natural diets (38.31 and 37.86 d, respectively) (Table 3). There was a significant difference in doubling time (DT); it was shorter (6.65 d) on the artificial diet than on the natural diet (7.96 d). There was no significant difference in survival rate (S), birth rate (B), and death rate (R) between the artificial and natural diets (Table 3).

**Table 3:** Impact of artificial and natural diets on the reproductive and population parameters of *P. gossypiella.*

Traits	Artificial diet	Natural diet
*r*/d	**0.10**	0.09
λ/d	**1.11**	1.09
*GRR*/offspring	**67.54**	33.49
*R*_*0*_ (offspring/individual)	**54.16**	27.17
Mean Generation Time (*T*)/d	**38.31**	37.86
Doubling Time (*DT*)/d	6.65	**7.95**
Survival Rate (*S*) at SASD/d	0.99	**0.10**
Birth Rate (*B*) at SASD/d	**0.12**	0.10
Death Rate (*R*) at SASD/d	0.01	0.01

*r*: intrinsic rate of increase, λ: finite rate of increase, *GRR*: gross reproductive rate (offspring), *R*_0_: net reproductive rate, SASD: stable age-stage distribution; d: day. Higher mean values are represented in bold.

### Survival rate

**Figure 1:**
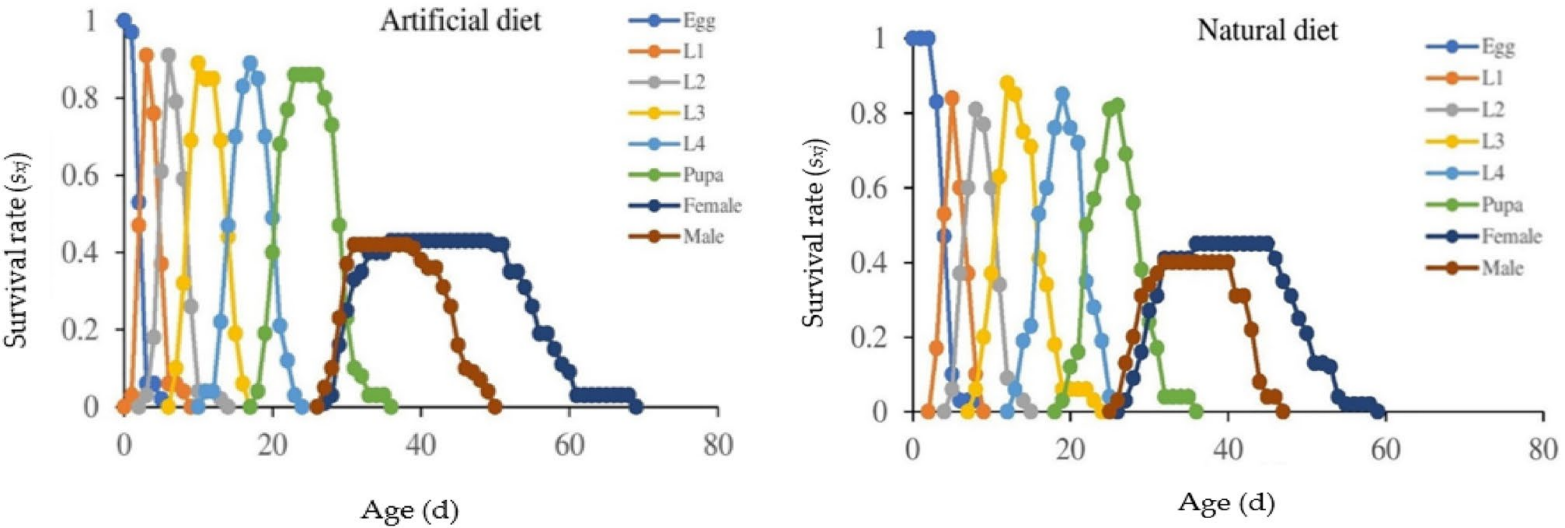
Impact of artificial and natural diets on the age-stage-specific survival rate (*s*_*xj*_) of the *P. gossypiella*. L1: 1st instar larvae, L2: 2nd instar larvae, L3: 3rd instar larvae, L4: 4th instar larvae.

The comprehensive age-stage survival rate (*s*_*xj*_) of *P. gossypiella* on different diets was determined (Fig. 1). Findings showed the possibility of a freshly hatched larva surviving to age *x* and stage *j.* Because developing rates varied across individuals on different diets, substantial differences in intersecting plotted graphs for different developmental phases were also noted (Fig. 1). The projected curves exhibited completely different layouts at each developmental stage. Individual survival rates rapidly dropped as age increased, and it showed an inverse relationship between survival and age (Fig. 1). The developmental time of female adults of *P. gossypiella* was longer on the artificial diet (36–49 d) in comparison to the natural diet (36–45 d), while the survival rate of female adults was shorter (0.43 *s*_*xj*_) on the artificial diet in comparison to the natural diet (0.45 *s*_*xj*_). In the case of male adults, the developmental time was shorter (31–38 d) on the artificial diet in comparison to the natural diet (32–40 d). The survival rate of male adults was higher on the artificial diet (0.42 *s*_*xj*_) than on the natural diet (0.40 *s*_*xj*_) (Fig. 1).

### Age-specific fecundity

**Figure 2:**
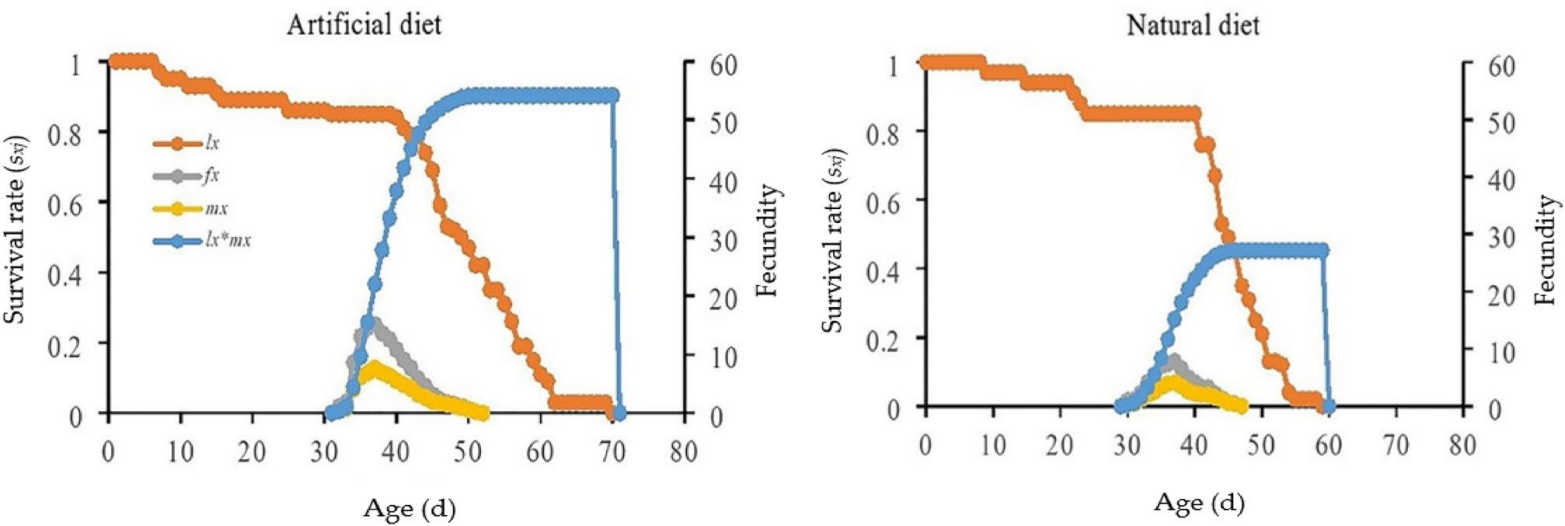
Impact of artificial and natural diets on the age-specific survival rate (*l*_*x*_), female age-specific fecundity (*f*_*x*_), age-specific fecundity (*m*_*x*_), and age-specific maternity (*l*_x_*m_*x*_) of the *P. gossypiella*.

The peak noted value of age-stage specific fecundity (*f*_*xj*_*)* was higher on the artificial diet compared to the natural diet (Fig. 2). There was a direct relation in age-specific maternity (*l*_x_*m_*x*_) of the *P. gossypiella* (Fig. 2). As survival rate increased, fecundity also increased in both cases (artificial and natural) (Fig. 2). The constant peak points of age-specific maternity (*l*_x_*m_*x*_) of the *P. gossypiella* were 54.15 eggs/d from 50 to 69 d, which was higher than the natural diet (27.16 eggs/d from 46 to 59 d) (Fig. 2). The *m*_*x*_ curves indicated that reproduction started comparatively earlier on the natural diet than the artificial diet. The highest value of age-specific fecundity (*m*_*x*_) was higher on the artificial diet than on the natural diet (Fig. 2).

### Life expectancy

**Figure 3:**
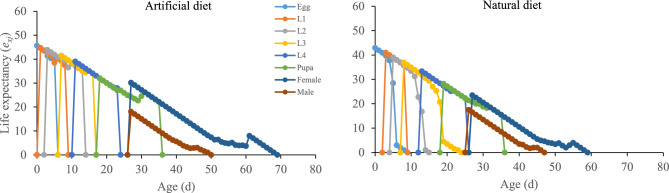
Stimulus of artificial and natural diets on the age-stage-specific life expectancy (*e*_*xj*_) of the *P. gossypiella*. L1: 1st instar larvae, L2: 2nd instar larvae, L3: 3rd instar larvae, L4: 4th instar larvae.

The effects of different diets on the population’s life expectancy (*e*_*xj*_) at each stage of *P*. *gossypiella* were determined (Fig. 3). The longevity of the newly hatched *P*. *gossypiella* eggs was longer on the artificial diet compared to the natural diet (Fig. 3). The *e*_*xj*_ was 45.62 d, which was higher than that on the natural diet (42.92 d) (Fig. 3). The peak value of *e*_*xj*_ in female adults of *P. gossypiella* was maximum on artificial diet in comparison to natural diet. Overall, higher *e*_*xj*_ of all stages of the *P. gossypiella* were observed on the artificial diet than on the natural diet (Fig. 3).

### Reproductive value

**Figure 4:**
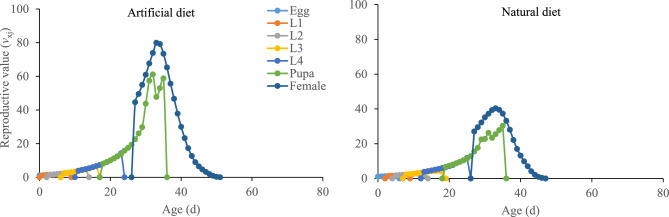
Impact of artificial and natural diets on the age-stage reproductive value (*v*_*xj*_) of the *P. gossypiella*. L1: 1st instar larvae, L2: 2nd instar larvae, L3: 3rd instar larvae, L4: 4th instar larvae.

Age-stage reproductive value (*v*_*xj*_) explains an individual’s contribution to the future population (i.e., the population forecasting scale) at age *x* and stage* j*. Results showed that reproductive value *v*_*xj*_ significantly increased (79.92 *v*_*xj*_ on 33 d) on the artificial diet and also increased (40.37 *v*_*xj*_ on 33 d) on the natural diet. However, the results revealed that the age-stage reproductive value (*v*_*xj*_) of the *P. gossypiella* is higher on the artificial diet than on the natural diet. The artificial diet had more positive effects on the reproductive value (*v*_*xj*_) of *P. gossypiella* (Fig. 4).

### Age distribution mortality (Matrix p)

**Figure 5:**
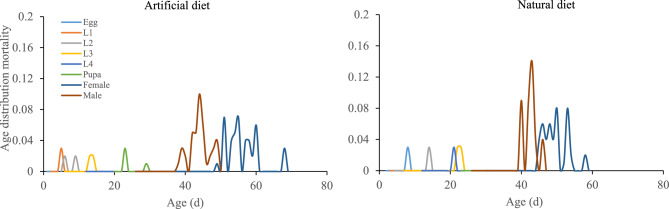
Impact of artificial and natural diets on the age distribution mortality (matrix p) of the *P. gossypiella*. L1: 1st instar larvae, L2: 2nd instar larvae, L3: 3rd instar larvae, L4: 4th instar larvae.

The age distribution mortality of each stage of *P. gossypiella* was higher on the natural diet than on the artificial diet as shown in Fig. 5.

## Discussions

The biology/fitness of *P. gossypiella* has been covered on various natural host plants and cotton, corn and wheat based artificial diets using old/simple table analysis in a number of studies^28,33–35^. When study the biology of an insect on artificial diet as compared to growing on natural food, feeding promote fitness reduces the amount of work, time, space, and money needed to cultivate the host plants. Additionally, the availability of artificial food enables easier agreement of insect growth^36^. In the case of *P. gossypiella*, providing natural hosts all year around is difficult. As an alternative to natural diet, observing the fitness of *P. gossypiella* can be more convenient with alternative diet*.* Study of fitness parameters via two-sex life table is the most effective for describing the age-stage dynamic of both male and female insects^30,31,37^: as compared to old life table tools that only give the data on female adults only and cannot explain the age-stage parameter of male and female insects, while the Two-sex life table parameters of *P. gossypiella* had not been used on cotton-seed based artificial diet. Therefore, the two-sex life table features of *P. gossypiella* were described in our work for the first time.

Food plays the most important role in the growth and developmental rate of the *Bactrocera dorsalis* (Diptera: Tephritidae)^38,39^. Proteins and other nutrients help insects’ development, especially enhancement of insect fecundity, which is why they are important nutritional elements in diets used to mass-raise insect pests in laboratories^40,41^. Female adults of bollworms that were fed on any of the artificial diets, regardless of the protein, are unable to produce viable and sufficient eggs^42^. In our study, cotton seed-based artificial diets enhanced the fecundity and fitness of *P. gossypiella.* Because cotton seeds are rich source high-quality protein and nutrients^29^.

The incubation period on the artificial diet was shorter than on the natural diet (Table 1). This finding was in contrast to the results of Jothi et al. (2016), who reported a longer incubation period of *P. gossypiella* because of diet ingredients, e.g., cotton seed flour and chickpea flour (processed)^43^. Diet constituents are more important in enhancement of incubation period as well as eggs. The survival of bollworms is more negatively impacted by protein less diets than as compared to carbohydrate deficits. Similarly, another study revealed that the incubation period of *P. gossypiella* was shorter i.e., 4.9 days on artificial food^35^ using the same ingredients and procedure as described by the authors of^43^. Another study showed that the incubation period of *P. gossypiella* was 4 days on a natural diet (cotton bolls) and 3.89 days when fed on an okra diet^44^. So, these studies reported shorter incubation period of *P. gossypiella.* These results are in contrast to present findings because we used a different controlled conditions (27 ± 2 °C and 65 ± 5% RH) and cotton-seed based diet that is enrich in protein^42^.

The larval duration of *P. gossypiella* was in the range of 18–21 days on a chickpea flour- and wheat-gram-based diet^28^. However, in another study it was reported that the average larval duration of *P. gossypiella* was 25 days on an artificial diet^43^. These findings were different to the present study due to different rearing techniques and, mainly, difference of artificial diet ingredients. While on a natural diet, the larval period of *P. gossypiella* was reported to be about 22 days^24^, which is close to our results of larval duration when fed on mature cotton bolls.

Pupal duration of *P. gossypiella* was in the range of 8–9 days on an artificial diet^26,28,45^, while another study revealed that the pupal period of *P. gossypiella* was 16 days^46^. Despite the natural diet, the pupal developmental period of *P. gossypiella* has been reported as 9–10 days on a natural diet^25,27^, Pupal development and fecundity are important parameter for insect development and fitness^47,48^. while in our study the pupal duration was longer on the artificial diet in comparison to the natural diet. In this study, the fecundity of female *P. gossypiella* adults was higher on artificial diets. Female adult insects often live longer than male adults, according to the previous literature, which highlights the difference in longevity between the sexes^24,28,44^. Similarly, another study reported that females of *Earias vittella* (Lepidoptera: Noctuidae) lived longer than males when fed on cotton bolls and okra^49^. Similar results were observed on both foods in this study.

Oviposition period in insects is a significant component in understanding species’ presence and population levels^50^. In the current study, we found that the oviposition period of *P. gossypiella* was highest on the artificial diet in comparison to the natural diet. A different study reported that the oviposition period of *P. gossypiella* was almost similar to the current study^35^, while another study revealed that the oviposition period of *P. gossypiella* was shorter than that in the current study because of differences in diet components^25^. Insects’ ability to produce healthy larvae or nymphs depends on when they ovulate and their eggs hatch^51^. Different species have different fecundity rates^52^. Egg laying in insects has a significant effect on the dynamics of the insect population^48^. Fecundity depends on the insect food^53^. In our results, we found higher fecundity and longer oviposition period of *P. gossypiella* when fed on the cotton based artificial diet.

Two-sex life table studies help us understand the ecology and fitness of an organism since they account for both sexes while building the right population curve for future populations which will help researcher in designing pest management strategies against any pest^31^. When defining how food affects an insect pest’s fitness, reproductive traits *r*, *GRR λ*, and *R*_*0*_ values are crucial^54^. In order to represent how an insect has adapted to a food supply, *r* may be the most appropriate population index if it is greater than *0*. In our study, *P. gossypiella’s* artificial diet with a cotton base resulted in lower mortality and increased rates of reproduction and birth. The immature life span of *P. gossypiella* did not appear to differ for either diet. Results concluded that the fecundity of *P. gossypiella* was highest on the cotton seed-based artificial diet as compared to natural diet. Because cotton bolls were not sufficient diet for larvae of *P. gossypiella* in laboratory environment. Future work on the *P. gossypiella* in laboratory and fields will be benefit from our study.

## Methodology

### Mass production

The *P. gossypiella* population (larvae and pupae) was collected from several cotton fields of Khanewal and Kacha Khu (Location: 30° 26′ 11″ N and 72° 13′ 55″ E, respectively). Larvae collected from infested rosette flowers and infested cotton bolls were kept in plastic jars (0.09 × 0.09 × 0.12 m) with cotton seed based artificial diet for pupation covered with muslin cloth. Pupae were placed into a Petri dish of 0.09 m diameter and placed into a cage (0.25 m × 0.25 m × 0.12 m). Adults food containing cotton swabs soaked in aqueous solution of honey and vitamins in equal ratio were given into the cage. Adult diet source were changed daily. After 12 h, cotton shoots about 0.07 m long were fitted in a test tube (0.37 × 1 m) containing water and placed inside the cage for egg laying. The adults of *P. gossypiella* laid fertilized eggs on shoots having flowers and leaves spread in the cage. Eggs of pink bollworm are whitish green color. Two stock cultures of *P. gossypiella* were maintained in the laboratory conditions (27 ± 1 °C, 65 ± 5% R.H.) separately on dissected locules of mature cotton boll and artificial diet. Both of cultures were raised at least for two generations to acclimatize on food and in laboratory conditions.

### Life traits on natural diet

One hundred eggs of *P. gossypiella* laid on small cotton shoots were collected and allowed to hatch. After egg hatching (2–4 days), a jar was placed upside down on a table. A piece of white paper was placed on the table to observe the 1st instar larvae. Newly hatched larvae were fed singly on a locule of non-Bt cotton (CIM-717) bolls. All bolls were washed with simple water followed by drying with tissue paper for 5 min. After that, cotton bolls were placed in a jar containing 0.1% formalin solution (for 2 min), following the methodology of Fand et al.^24^. All bolls were washed again with simple water. A small hole measuring 1 mm in diameter was made in each cotton boll with the help of needle. Holes should be about 1–2 cm long so larvae can easily live in the burrow-like holes and also feed with ease. First instar larvae were put through the holes into the cotton bolls. When larvae hatch from eggs, both of male and female’ first instar larvae are white in color having brown head. Third instar larvae have visible pink marks with creamy to ivory body. Larvae went through four growth stages (instars), starting to turn pink in the fourth instar. While, the male larvae (in 3rd instar) have two light spots on dorsal side of the abdomen, while in 4th instar these two spots becomes darker. One hundred larvae were observed till adult formation. Mature cotton bolls were replaced after 2 days. The bolls were first gently washed with tap water to remove any inherent dirt, then disinfected by dipping in a 0.1% formalin solution for two to three minutes to prevent any secondary infections, further rinsed with tap water to remove any remaining formalin, and finally shade dried in an open space. Each jar contained one cotton boll and was covered with muslin cloth. Larval instars were checked after every two days by opening the cotton boll with sharp knife. Cutting is done in the line of locule that was totally safe for immature of *P. gossypiella.*

Pair of male and female pupae of *P. gossypiella* were separated and placed into the jars (0.18 × 0.18 × 0.16 m)^55^. The cotton shoots about 0.07 m long (for egg laying) were fitted in a test tube (0.37 × 1 m) containing water and placed inside the cage; replaced daily after counting eggs under the stereo microscope. In total, 10 pairs were used to observe the reproductive parameters, i.e., mating, pre-oviposition, oviposition, and fecundity. Data were recorded on a daily basis until the death of the male and female parents.

### Life traits on the artificial diet

The cotton seed-based artificial diet was prepared following the methodology of Ihsan et al.^28^, with some modifications as follows: fraction (A): cotton seed flour, chickpea flour, and sucrose were mixed in 200 mL water, fraction (B): agar–agar was boiled in water with continuous stirring, and fraction (C): basin salt, streptomycin sulphate, dried yeast powder, methyl 4-hydroxy benzoate, multivitamin, casein, cystiene, wesson salt, ascorbic acid, and sorbic acid were mixed. After preparation of these three fractions, they were mixed together in a plastic tray and put in a refrigerator. The exact quantity of diet components was measured using an electronic weight balance as mentioned in Table 1.

One hundred eggs from acclimatized population of *P. gossypiella* that were reared on artificial diet were collected. Newly hatched larvae were singly released on artificial diet in petri-dishes of 0.5 m diameter each covered with lids having aeration holes. The lids were sealed with the help of parafilm warped around. Diet was replaced after every 48 h. Data of life table traits regarding larval instars and pupal duration were recorded daily. The pupae were placed in glass jars and moths emerged were allowed to mate (Supplementary material). Ten pairs of *P. gossypiella* were isolated and kept in glass jars with liquid food. Some important life traits like durations of mating, pre-oviposition and oviposition along with fecundity were recorded.

### Statistical analysis

Means of each biological parameter for *P. gossypiella* were analyzed in the SPSS Statistics 15.0. The TWOSEX program was used to assess the *P. gossypiella,* age-stage-specific survival rate (*S*_*xj*_), age-stage life expectancy (*e*_*xj*_), age-specific fecundity (*m*_*x*_), age-specific survival rate (*l*_*x*_), and age-stage reproductive value (*v*_*xj*_), as well as criteria for the life table (finite rate of increase r, net reproductive rate *R*_*0*_, mean generation time *T*, and number of stages *k*)^56^.

Equations of *l*_*x*_*, m*_*x*_*,* and* R*_*0*_ are as follows,

1 $$ l_{x} = \mathop \sum \limits_{j = 1}^{k} S_{xj} $$

2 $$ m_{x} = \frac{{\mathop \sum \nolimits_{j = 1}^{k} S_{xj } f_{xj} }}{{\mathop \sum \nolimits_{j = 1}^{k} S_{xj} }} $$

3 $$ R_{0} = \mathop \sum \limits_{x = 0}^{\infty } l_{x} m_{x} $$

where* k* is no. of stages. In this study, the Euler–Lotka formula’s iterative bisection approach was used to estimate the *r*, with the age index starting at 0 as in Eq. (2)^57^.

4 $$ \mathop \sum \limits_{x = 0}^{\infty } e^{{ - r\left( {x + 1} \right)}} l_{x} m_{x} = 1 $$

5 $$ e_{xj} = \mathop \sum \limits_{i = x}^{\infty } \mathop \sum \limits_{y = j}^{k} s{^{\prime}}_{iy} $$

6 $$ V_{xj} = \frac{{e^{{r\left( {x = 1} \right)}} }}{{S_{xj} }}\mathop \sum \limits_{i = x}^{\infty } e^{{ - r\left( {x + 1} \right)}} \mathop \sum \limits_{y = j}^{k} s{^{\prime}}_{iy} f_{iy} $$

Finally, the bootstrap method was used to determine two-sex life table traits^58^. Using the TWOSEX-MS-Chart application, 200,000 new samples of the bootstrap method were run^37^.

### Supplementary Information

Supplementary Information.

## Data Availability

All data generated and analysed during this study are included in this published article and its supplementary information files.
