# Author Correction: Influence of natural and non‑natural diets on the fitness and rearing of *Pectinophora gossypiella* Saunders

**DOI:** 10.1038/s41598-023-47960-6

**Published:** 2023-11-23

**Authors:** Rabia Saeed, Muhammad Waqar Ul Hassan, Waqar Jaleel, Muhammad Ikhlaq, Syed Ishfaq Ali Shah, Safia Niaz, Rashid Azad, Rasheed Akbar, Zahid Mahmood, Adeel Mukhtar, Syed Muhammad Zaka, Khawaja G. Rasool, Mureed Husain, Montaser M. Hassan, Abdulrahman S. Aldawood, Muhammad Shakeel

**Affiliations:** 1 Entomology Section, Central Cotton Research Institute, Multan, Punjab 60000 Pakistan; 2 Department of Entomology, Faculty of Agricultural Sciences and Technology, Bahauddin Zakariya University, Multan, 60800 Pakistan; 3 Horticultural Research Station Bahawalpur, Multan, Punjab Pakistan; 4 Department of Agricultural Engineering, Fareed Biodiversity and Conservation Centre, Khawaja Fareed University of Engineering and Information Technology, Rahim Yar Khan, Punjab Pakistan; 5 Department of Entomology, Faculty of Basic and Applied Sciences, The University of Haripur, Haripur, Khyber Pakhtunkhwa 22062 Pakistan; 6 Institute of Environment and Ecology, School of the Environment and Safety Engineering, Jiangsu University, Zhenjiang, China; 7 Plant Protection Department, College of Food and Agriculture Sciences, King Saud University, P.O. Box 2460, 11451 Riyadh, Saudi Arabia; 8 Key Laboratory of Bio‑Pesticide Innovation and Application of Guangdong Province, College of Agriculture, South China Agricultural University, Guangzhou, China; 9 Department of Biology, College of Science, Taif University, P.O. Box 11099, 21944 Taif, Saudi Arabia

Correction to: *Scientific Reports* https://doi.org/10.1038/s41598-023-40712-6, published online 22 August 2023

In the original version of this Article, Montaser M. Hassan was incorrectly affiliated with ‘Plant Protection Department, College of Food and Agriculture Sciences, King Saud University, P.O. Box 2460, 11451, Riyadh, Saudi Arabia.’

The correct affiliations are as follows:

Department of Entomology, Faculty of Basic and Applied Sciences, The University of Haripur, Haripur, Khyber Pakhtunkhwa, 22062, Pakistan.

Department of Biology, College of Science, Taif University, P.O. Box11099 Taif 21944, Saudi Arabia.

The original Article has been corrected.

